# Black Lives Matter… but to Whom? An Examination of Nationally-Grounded Determinants of Black Lives Matter Support

**DOI:** 10.5334/irsp.824

**Published:** 2023-11-20

**Authors:** Lucie-Anna Lankester, Theodore Alexopoulos

**Affiliations:** 1 Université Paris Cité, France; 2 Université de Bordeaux, France

**Keywords:** Black Lives Matter, Cultural norms of diversity, Laïcité, National Identification, Social Dominance Orientation

## Abstract

Black Lives Matter (BLM) has gained momentum in its fight against racism cross-nationally. Yet, there are conflicting opinions on BLM. To account for this, previous research relied on cross-national predictors (e.g., Social Dominance Orientation; SDO). However, BLM support needs to be examined via the prism of national contexts and their peculiarities. Here, we claim that nationally-grounded determinants (next to cross-national ones) shape BLM (dis-)approval. Based on national identity construal, we argue that the way individuals identify with it predicts BLM support. Further, we expect this relationship to be mediated by personal endorsement of national beliefs about diversity. This claim was tested in a French ecological context, where: 1) national identity is based on a straitened view of diversity and 2) diversity issues are regulated via two antagonistic cultural norms: one is egalitarian (i.e., Historic Laïcité) and the other is assimilationist (i.e., New Laïcité). In two pre-registered and well-powered studies (Study 1, *N* = 305; Study 2, *N* = 489), we anticipated and found that National Identification negatively predicts BLM support. Crucially, cultural norm endorsements dually-mediated this relationship, suggesting their instrumental function in BLM support. We considered, via path analysis, an additional pathway involving SDO. We found that nationally-grounded and cross-national paths operate jointly to shape BLM support. We replicated these results one year later, providing support for our model. These findings are of relevance beyond the French context as they contribute to an emerging literature examining how intra- and inter-national forces shape, in tandem, diversity responses.

The Black Lives Matter (BLM) movement—denouncing systemic anti-Black racism in the United States (US)—has been characterized as the ‘*civil rights issue of our time*’ (Chase, 2018; Holt, 2018). In May 2020, after a White police officer killed a Black man (George Floyd), BLM protests spread across the United States and then to other countries (e.g., France, Brazil; Time Magazine, 2020). Research initially focused on cross-national determinants to explain differences in BLM support (e.g., Social Dominance Orientation, SDO; Holt, 2018; Holt & Sweitzer, 2020; Sidanius & Pratto, 2001). However, to our knowledge, BLM support has never been examined at a national level through the prism of its internationalization. Yet, the BLM protests are encapsulated within national contexts, where specific determinants should drive the movement’s acceptance. Granting this, in the present paper, we investigate whether nationally-grounded variables, next to cross-national ones, contribute unique variance in BLM support. In doing so, we contribute to the growing literature arguing that the national context is often a neglected force when examining diversity responses (Guimond et al., 2013; Pehrson et al., 2009; Wagner et al., 2021; Weldon, 2006).

Nations represent interlaced matrices of situated political and cultural forces inside which diversity issues, such as the BLM, are addressed. Crucially, at a national level, national identity content determines how different ethno-cultural groups are included within the larger national group (Yogeeswaran & Dasgupta, 2014). In turn, individuals’ level of self-affiliation with this identity (i.e., National Identification; Staerklé et al., 2010) shapes their diversity responses (Pehrson et al., 2009; Roccas et al., 2006). Moreover, each nation relies on *cultural norms of diversity* specifically aimed at managing cultural particularisms within social settings (Guimond et al., 2013). Research shows that individuals use these norms to sustain their personal motives (e.g., ties to national identification) when dealing with diversity issues (Adam-Troian et al., 2019; Badea, 2012; Badea et al., 2018). As such, National Identification and its ancillary cultural norms of diversity represent crucial national-cultural building blocks on which BLM (dis-)approval is based.

France appears as a highly relevant national context to test this rationale. Indeed, France rests on a national identity blind to group-based identities, which *de facto* opposes the BLM’s narrative (Goldman, 2021; Kamiejski et al., 2012b).What’s more, to address diversity issues, individuals can mobilize two distinct cultural norms of *Laïcité* (i.e., French secularism): The *Historic Laïcité* (i.e., a long-standing egalitarian norm), and the *New Laïcité*, (i.e., a recently amended version of the former oriented toward assimilation; Lankester & Alexopoulos, 2021; Roebroeck & Guimond, 2017). Based on these elements, we examine whether French National Identification reliably predicts BLM (dis-)approval. Further, we anticipate that the level of endorsement of both Laïcité will mediate this relationship (Adam-Troian et al., 2019; Badea, 2012; Badea et al., 2018). Additionally, to further substantiate our claim, we examine an additional pathway starting with SDO. We conducted two well-powered and pre-registered studies, one at the peak of the protests and a close replication one year later, to assess whether the suggested model is reliable in a different context and time frame.

## BLM Support in France: How National Identification Matters

Across nations, National Identification is a key driver of the support for minorities’ rights, the fight against discrimination and prejudice (Banfield & Dovidio, 2013; Pehrson et al., 2009; Verkuyten, 2009; Yogeeswaran & Dasgupta, 2014). Yet, National Identification predicts contrasting diversity responses depending on the shared conceptions of national identity (Brewer, 1999; Pehrson et al., 2009; Weldon, 2006).^1^ Indeed, at a national level, National identities can be classified as ‘exclusive’ (i.e., promoting the native national group) or ‘inclusive’ (i.e., acknowledging cultural diversity; Pehrson et al., 2009). Importantly, when national identity is ‘exclusive’ (vs. ‘inclusive’), diversity responses are more negative (Hopkins, 2001; Pehrson et al., 2009; Weldon, 2006). France represents a prime example of this type of relationship. National identity is molded around the idea that ‘being French is the only significant identity category—not religion nor race nor ethnicity’ (Beaman & Petts, 2020: 5). For instance, minorities are required to abandon their cultural affiliations especially in public settings (Kamiejski et al., 2012b). As such, French national identity is generally viewed as being ‘exclusive’ (Badea, 2012; Kamiejski et al., 2012a; Ocak, 2016; Simon, 2012, 2013).^2^ Significantly, higher French National Identification predicts negative diversity responses (Adam-Troian et al., 2019; Badea, 2012; Badea et al., 2018; Da Silva et al., 2021; Pehrson et al., 2009; Weldon, 2006).

Following this rationale, in France, the BLM protest’s spotlighting of systemic racism (and highlighting those targeted by it) seems at odds with ideals of national identity (Goldman, 2021; Kamiejski et al., 2012b). For instance, during a BLM protest, French ultra-nationalists hoisted a ‘White Lives Matter’ banner (France Info Television, 2020). Besides the radical nature of this event, it suggests that high national identifiers may dismiss the BLM movement to protect their national identity, values, and culture (Adam-Troian et al., 2019; Badea & Aebischer, 2017; Grajzl et al., 2018; Stephan & Stephan, 2000; Verkuyten & Yildiz, 2006). Therefore, we expect National Identification to negatively predict BLM support in France. Further, we contend that the endorsement of cultural norms of diversity accounts for this relationship (Adam-Troian et al., 2019; Badea, 2012; Badea et al., 2018).

## Diversity Norms as Functional Arguments to Legitimize BLM Support

National identity defines who belongs to a specific nation. Additionally, cultural norms of diversity direct how minority groups should display their cultural differences to fit in.^3^ There is growing evidence that these norms serve an instrumental function regarding National Identity concerns (Adam-Troian et al., 2019; Badea, 2012; Badea & Aebischer, 2017; Badea et al., 2018; Gieling et al., 2014; Grajzl et al., 2018). Crucially, when national identity is ‘exclusive’, national identifiers tend to support assimilationist norms (i.e., summons to suppress diversity affiliations) and oppose multiculturalist norms (i.e., valuing diversity identities; Badea, 2012; Badea et al., 2018). Moreover, assimilation endorsement drives prejudicial responses, while multiculturalism endorsement leads to more favorable diversity responses (see Whitley & Webster, 2019, for a meta-analysis). Consequently, the support for specific cultural norms has been suggested to act as a potent mediator between National Identification and diversity responses (Adam-Troian et al., 2019; Badea, 2012; Badea & Aebischer, 2017; Badea et al., 2018).

Again, France provides a unique opportunity to examine this, because there is not one, but two dominant cultural norms. Indeed, due to galloping socio-political changes, the French cultural norm, the *Laïcité*, is promoted in two distinct forms (Roebroeck & Guimond, 2017). The Historic Laïcité (i.e., its original form), is an egalitarian norm used to fend off discrimination rooted in ethno-religious particularities. The New Laïcité (i.e., its recent amended form) is an assimilationist norm fostering social uniformity in public settings (for a review, see Lankester & Alexopoulos, 2021). Interestingly, both Laïcité norms are associated with contrasting responses toward diversity issues that go well beyond the religious domain they were originally intended to address. For instance, endorsing New Laïcité predicts more negative responses toward minority groups (i.e., Maghrebians and foreigners) than endorsing Historic Lacité (Anier et al., 2018; Kamiejski et al., 2012b; Roebroeck & Guimond, 2017). Based on this, one could expect that they drive distinct responses toward societal diversity issues such as the BLM protest.

What is more, a recent study shows that New Laïcité endorsement mediates the link between National Identification and diversity responses (Adam-Troian et al., 2019). Yet, the present contribution goes one step further by considering the concurrent functional role of both norms. Specifically, the New Laïcité’s focus on assimilation seems to better address the concerns surrounding the French national identity as compared to the Historic Laïcité’s egalitarian core (Adam-Troian et al., 2019; Kamiejski et al., 2012b). Thus, we expect Higher National Identifiers to favor the New Laïcité and to discard the Historic Laïcité (Badea et al., 2018). In turn, the New Laïcité may set the ground to justify the dismissal of the protest (e.g., ‘displaying ethnic identities goes against the Laïcité’), while the Historic Laïcité may function as a ground to support it (e.g., ‘the Laïcité serves to curb systemic racism’).

## An Additional Path Stemming From a Cross-Cultural Determinant

To better gauge the variance in BLM support explained by nationally-grounded variables, we also considered a cross-national determinant. In fact, to account for the diverging reactions to BLM, some research relied on Social Dominance Theory (SDT; Sidanius & Pratto, 2001). According to SDT, societies are structured in group-based hierarchies where dominant groups enjoy more privileges than subordinate groups. Individuals vary in their degree of support for this hierarchy, captured by the notion of Social Dominance Orientation (SDO). High-SDO individuals report more negative diversity responses, especially to reaffirm the established group hierarchy. For instance, research conducted in the US context suggests that SDO negatively predicts BLM support (Holt, 2018; Holt & Sweitzer, 2020; Solomon & Martin, 2019). Thus, SDO should to some extent predict BLM (dis-)approval across nations, including France (Sibley & Duckitt, 2008). However, regarding BLM support, studies neglected a fundamental aspect of SDT: the link between SDO and diversity responses is accounted for by legitimizing myths (i.e., beliefs that legitimate one’s SDO level; Sidanius & Pratto, 2001). Interestingly, cultural norms of diversity represent such legitimizing myths (Guimond et al., 2013; Hindriks et al., 2014; Levin et al., 2012).

In the French context, Laïcité norms’ endorsement has been considered through this lens. The rationale holds that high SDO individuals are likely to favor the New Laïcité to bolster the status quo, while low SDO individuals tend to marshal the Historic Laïcité to offset inequalities (Kamiejski et al., 2012b; Roebroeck & Guimond, 2017; Troian et al., 2018). As discussed above, this differential endorsement of the two Laïcité predicts, in turn, contrasting diversity responses (Kamiejski et al., 2012b; Roebroeck & Guimond, 2017, 2018). However, to our knowledge, a dual-mediation test to establish their legitimizing myth’s function is still needed (see Lankester & Alexopoulos, 2021). Thus, the inclusion of an additional path starting with SDO fills this gap. What is more, it contributes to shed new insights on the fundamental variables explaining the relationship between SDO and BLM support.

## The Present Research

Taking France as a key diagnostic specimen, we examine whether nationally-grounded variables shape BLM support. We expect that National Identification will negatively predict BLM support and that Laïcité endorsement will dually mediate this relationship: the more French are national identifiers, the more they will endorse the assimilationist New Laïcité, and the less they will favor the egalitarian Historic Laïcité, which, in turn, will drive less BLM support. Moreover, to examine whether this nationally-grounded pathway contributes unique variance in BLM support, we consider a pathway starting with SDO (i.e., a robust cross-national factor). Using path analysis, we jointly assess a) the indirect path from National Identification, via Laïcité endorsement, to BLM support, and b) the indirect path from SDO, via Laïcité endorsement, to BLM support. Based on previous research, we expect both indirect paths to operate jointly in shaping BLM support (Adam-Troian et al., 2019; Kamiejski et al., 2012b; Roebroeck & Guimond, 2017, 2018; Troian et al., 2018). Additionally, we conjecture National Identification to be a more potent predictor of Laïcité endorsement than SDO, as these constructs are inextricably intertwined in the French context to oppose/support BLM (Adam-Troian et al., 2019; Goldman, 2021; Mallard, 2020). We tested these hypotheses in the ecological context of the outburst of the social movement, running a first study amidst the peak period of the French BLM (i.e., June–July 2020). Furthermore, to assess the reliability of the postulated paths in another context and point in time, we conducted a replication one year later.

## Study 1

In the first study, to afford a crucial test of our rationale we capitalized on the ecological context of the French BLM protests which took place during the spring of 2020. All presented analyses were pre-registered and available at: Pre-registration Study 1.

### Power Analysis and Sample Size

Initially, we ran and preregistered a power analysis to detect an effect size of *η^2^* = 0.03 with 80% power based on the path between National Identification and diversity responses estimate in Badea (2012)’ study. This analysis yielded a required sample size of 242 participants, rounded up to 300 to avoid overestimation of the true population effect size (Perugini et al., 2018). However, afterwards we realized that Monte Carlo simulations were more suited to estimate the sample size for a dual mediation and path analysis (Beaujean, 2014; Muthén & Muthén, 2002). Thus, for both analyses, we conducted two additional Monte Carlo simulations. For each path, the parameter values were set by averaging the standardized coefficients reported in the relevant literature, with *n* = 300 (sample size) and *m* = 500 (number of samples) using the ‘simsem’ R package. These simulation analyses confirmed that the sample size is sufficient to reliably detect all regression coefficients with satisfactory power (>80%, for details see, supplementary material).

In total, 382 participants took part in the study implemented in Qualtrics and disseminated via social media. We excluded observations who had completion times below 3 minutes (*N* = 5).^4^ Moreover, in France, ethnic statistics are prohibited by law. Therefore, to secure a sample composed of French native born/speakers, we used participants’ mother tongue and education country as proxies for ethno-cultural membership. Thus, we excluded participants which either: did not complete their primary/secondary education in France (*N* = 22), reported both parents’ mother tongues other than French (*N* = 7), or related to North- and West-African tongues (*N* = 35).^5^ We thus analyzed the data of the remaining 305 participants: 187 women, 116 men, and 2 non-specified (age range: 18–87 years old; *M_age_* = 31.38; *SD_age_* = 17.58).

### Materials and Procedure

All materials, data, procedure, and complementary material can be found at: supplementary material. Participants initially completed the National Identification and SDO scales (the order of these scales was counterbalanced), then the Laïcité endorsement scales and finally the BLM support items. Unless otherwise specified, all items were assessed on 7-point Likert-type scales anchored at 1 (*strongly disagree*) and 7 (*strongly agree*).

#### National Identification

Four items (Badea, 2012) were used (e.g., ‘Being French is an important part of my identity’) and averaged into a single French National Identification index (*α* = .75).

#### Social Dominance Orientation

The short 8-item SDO scale (Duarte et al., 2004; Ho et al., 2015) was used (e.g., ‘Some groups of people are inferior to other groups’) and averaged into a single SDO index (*α* = .89).

#### Laïcité Endorsement

The Laïcité endorsement scale (Roebroeck & Guimond, 2017) was used. Seven items assessed the Historic Laïcité endorsement (e.g., ‘I do not want the French to be defined in terms of either their origin or their religion’) and six items assessed the New Laïcité endorsement (e.g., ‘As much as possible, religious practices should be private and not public’). We ran an exploratory factor analysis (EFA) using the ‘psych’ R package with an extraction method and Oblimin rotation (i.e., to gain potential information on the Laïcité dimensions correlation, although they are expected to be orthogonal; Roebroeck & Guimond, 2017). Bartlett’s test of sphericity and the KMO statistic confirmed respectively that the correlation matrix was not random, and sampling was adequate. The overall scale had a good internal consistency (*α* = .73). The parallel analysis and the visual scree test suggested two orthogonal factors (*r* = .001) corresponding to the Historic and New Laïcité dimensions. Nevertheless, two Historic Laïcité items showed cross-loadings (i.e., secondary loading on the New Laïcité factor > .30; Howard, 2016) and one New Laïcité item had a low loading (i.e., < .40; Gana & Broc, 2018). Thus, they were removed from the analysis (see supplementary material, for details). In sum, five items composed the Historic Laïcité index (*α* = .68), and five items composed the New Laïcité index (*α* = .76).

#### BLM Support

Although the study was conducted during the peak of the protests, we added a brief description of BLM before items completions: ‘As a reminder, Black Lives Matter—which translates in French as ‘La vie des noirs compte’—is a movement against racism born in the United States’. Then, three items adapted to the French context were presented (Selvanathan et al., 2018): ‘To what extent do you support the current Black Lives Matter protests taking place within French society?’ on a scale anchored at 1 (*I strongly oppose*) and 5 *(I strongly support*), ‘To what extent do you show your support for Black Lives Matter on social networks? (e.g., Facebook, Twitter, etc.)’ on a scale anchored at 1 (*never*) and 5 (*very often*), and ‘How likely are you to support future anti-racist protests in France?’ on a scale anchored at 1 (*not at all likely*) and 5 (*definitely likely*). These were collapsed into a single BLM support index (*α* = .79).

#### Sociodemographic Information

Finally, participants indicated their age, gender, mother tongue and parents’ mother tongue, whether they had completed their education in France and their political orientation.^6^

### Results

Means, standard deviations, and correlations among all measures in study 1 are presented in Table 1.

**Table 1:** Means, standard deviations, and inter-correlations between the main variables in Study 1.


VARIABLE	*M*	*SD*	1	2	3	4

1. National Identification	4.99	1.26				

2. SDO	2.29	1.21	.30***			

3. Historic Laïcité	6.33	0.77	–.16**	–.53***		

4. New Laïcité	4.94	1.37	.31***	.25***	–.04	

5. BLM support	3.10	1.07	–.34***	–.69***	.35***	–.40***


*Note*: N = 305, * Indicates *p* < .05, ** *p* < .01, *** *p* < .001.

#### Mediation Analysis

**Figure 1:**
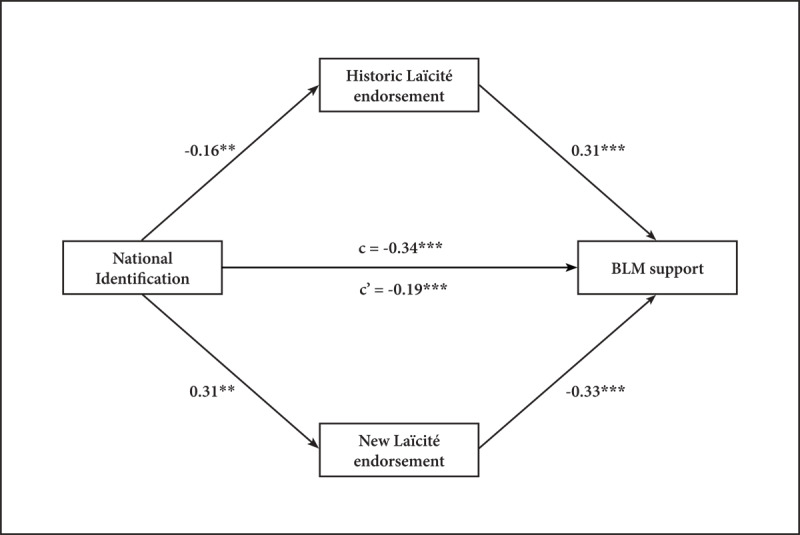
Mediation model of National Identification on BLM support via the endorsement of Laïcité norms. *Note*: coefficients are standardized in our sample (*N* = 305). c represents the total effect and c’ the direct effect of NI on BLM support. * Indicates *p* < .05; ** *p* < .01; *** *p* < .001.

To assess the dual-mediation, we followed the latest recommendations (i.e., component approach; Yzerbyt et al., 2018). We used the ‘mediate’ function from R package ‘psych’ and conduct 5,000 samples bootstrapped to estimate the indirect effect. As predicted, the results highlighted a mediation of National Identification on BLM support via Historic and New Laïcité endorsements (see Figure 1). The direct effect indicates that higher National Identification produces lower support for BLM, *b* = –0.19, *t*(301) = –3.69, *p* < .001. Moreover, the component paths are significant: National Identification predicts Historic Laïcité endorsement, *b* = –0.16, *t*(303) = –2.78, *p* = .005 and New Laïcité endorsement, *b* = 0.31, *t*(303) = 5.64, *p* < .001, and Historic Laïcité endorsement, *b* = 0.31, *t*(301) = 6.37, *p* < .001 and New Laïcité endorsement, *b* = –0.33, *t*(301) = –6.54, p < .001 predict BLM support. Finally, both indirect effects are significant: lower Historic Laïcité endorsement, *b* = –0.05, 95% CI [–0.09, –0.01] in combination with higher New Laïcité endorsement, *b* = –0.10, 95% CI [–0.15, –0.06] jointly mediate the effect of National Identification on BLM support.

#### Path Analysis

##### Hypothesized Model

**Figure 2:**
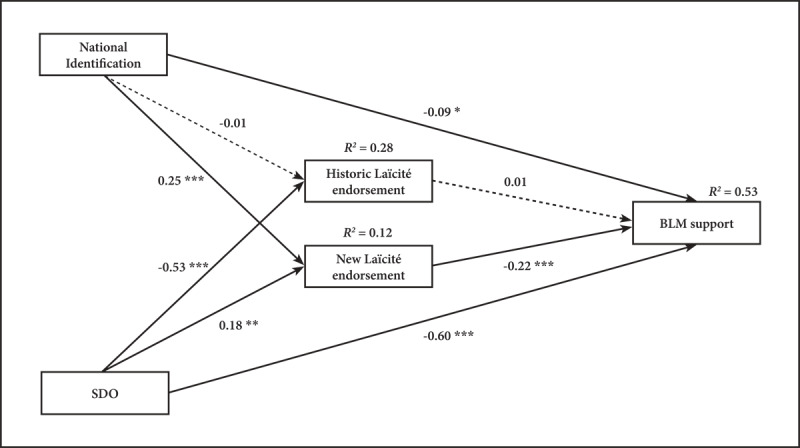
Path analysis including nationally-grounded and cross-national paths predicting BLM support. *Note*: Estimates path coefficients are standardized, and R^2^ indicates explained variance. Full lines represent significant paths and broken lines represent nonsignificant paths. * *p* indicates < .05; ** *p* < .01; *** *p* < .001.

To examine our Hypothesized model, National Identification and SDO were specified as predictors of both Laïcité endorsements. All four variables were specified as predictors of BLM support, and we also added the indirect paths.^7^ For this, we used the ‘sem’ function from the ‘lavaan’ R package and maximum likelihood estimation method (MLE; Gana & Broc, 2018). To assess the goodness of fit, we relied on several fit indices^8^ and examined two alternative models. The hypothesized model provided overall good fit to the data: CFI = 0.99, TLI = 0.92, SRMR = 0.02, AIC = 2272.31, except for RMSEA = 0.11, 90% CI [0.02, 0.21] (but see, Footnote 8). The model accounted for 53% of the variance of BLM support (R^2^ = 0.53). The results revealed a pattern of relationships mostly consistent with the predictions (see Figure 2).

To start with, BLM support is predicted by National Identification (*b* = –0.09, *p* = .03, 95% CI [–0.17, –0.01]), SDO (*b* = –0.60, *p* < .001, 95% CI [–0.68, –0.52]) and New Laïcité endorsement (*b* = –0.22, *p* < .001, 95% CI [–0.30, –0.14]), however, it is not linked to Historic Laïcité endorsement (*b* = 0.01, *p* = .82, 95% CI [–0.08, 0.10]). Furthermore, New Laïcité endorsement is predicted by National Identification (*b* = 0.25, p < .001, 95% CI [0.15, 0.36]) and SDO (*b* = 0.18, p =.001, 95% CI [0.07, 0.29]). Yet, Historic Laïcité is not predicted by National Identification (*b* = 0.001, *p* = .97, 95% CI [–0.09, 0.10]) while it is predicted by SDO (*b* = –0.53, *p* < .001, 95% CI [–0.61, –0.45]). Concerning the indirect pathway, as expected, the indirect path stemming from National Identification, via the New Laïcité, to BLM support is significant (*b* = –0.06, *p* < .001, 95% CI [–0.09, –0.02]), yet the one via the Historic Laïcité is not reliable (*b* < 0.001, *p* = .98, 95% CI [–0.001, 0.001]). The indirect pathway stemming from SDO via New Laïcité, to BLM support is also significant (*b* = –0.04, *p* = .005, 95% CI [–0.07, –0.012]) but the one via Historic Laïcité is not (*b* = –0.005, *p* = .82, 95% CI [–0.05, –0.04]). Finally, we performed a Wald test using the ‘lw’ function (Klopp, 2019) to compare the coefficients of the SDO-New Laïcité and National Identification-New Laïcité paths (*b* = 0.18 vs. *b* = 0.25, respectively). This test was not significant (Wald = 0.67, *p* = .41).

##### Alternative Models

We provide additional support for the Hypothesized model by testing it against two alternative models. In the Alternative model 1, we reversed the mediation of interest: the Historic and New Laïcité were now specified as predictors of National Identification and all three variables were specified as predictors of BLM support. Based on previous research, SDO was specified as a covariate of both Laïcité (Kamiejski et al., 2012b; Roebroeck & Guimond, 2017), and an independent predictor of BLM (Holt, 2018; Holt & Sweitzer, 2020). The overall fit indices are weaker than the hypothesized model: CFI = 0.98, TLI = 0.88 and SRMR = 0.05, AIC = 4242.34, RMSEA = 0.12, 90% CI [0.06, 0.20]. In the Alternative model 2, we considered the four variables as independent predictors of BLM support, but we allowed both National Identification and SDO to covary with Historic and New Laïcité (Adam-Troian et al., 2019; Kamiejski et al., 2012b; Roebroeck & Guimond, 2017). As expected, alternative model 2 had an overall poorer fit: CFI = 0.93, TLI = 0.67 and SRMR = 0.11, AIC = 4259.35, RMSEA = 0.20, 90% CI [0.13, 0.26]. Furthermore, Vuong’s test for non-nested models (Merkle et al., 2016) suggested that the hypothesized model is distinguishable from Alternative model 1 (w2 = 1.32, *p* < .001) and from Alternative model 2 (w2 = 1.36, *p* < .001). Moreover, the Hypothesized model fits the data better than the Alternative model 1 (z = 48.94, *p* < .001) and Alternative model 2 (z = 48.59, *p* < .001).^9^

### Discussion

Study 1 documents the first evidence, to our knowledge, that nationally-grounded determinants contribute unique variance to BLM responses. The more French are national identifiers, the less they support the BLM movement. Notably, this effect is dually mediated by the endorsement of French cultural norms: the (egalitarian) Historic and the (assimilationist) New Laïcité. Moreover, using path analysis, we found that both SDO and National Identification, via the New Laïcité, contribute independently to influence BLM responses. However, the two expected indirect paths involving Historic Laïcité endorsement are unreliable. This is surprising as prior research showed that, even after adjusting for SDO, Historic Laïcité endorsement accounts for a distinct portion of variance in diversity responses (Roebroeck & Guimond, 2017). One possible explanation could stem from measurement error of this construct that led to under/over-estimation of path coefficients (Cole & Preacher, 2014). Finally, the expectation that National Identification has a higher predictive strength than SDO on Laïcité endorsement is not met. Yet, to bring convergent evidence and test the reliability of our findings over time, we conducted a replication one year later.

## Study 2

The aim of the second study is to assess whether National Identification and Laïcité endorsement represent reliable predictors of French responses to BLM at another point in time. Notably, most scholars agree that over the past 15 years French national identity fosters unfavorable responses toward diversity issues (Adam-Troian et al., 2019; Badea, 2012; Badea & Aebischer, 2017; Badea et al., 2018; Da Silva et al., 2021; Pehrson et al., 2009; Weldon, 2006). Similarly, both Laïcité have been found to be reliable predictors of contrasting diversity outcomes across time (Anier et al., 2018; Kamiejski et al., 2012b; Roebroeck & Guimond, 2017). Therefore, we expected to replicate the dual mediation and path model with a second independent sample of French participants.^10^ We tested this rationale one year after Georges Floyd’s death, a period marked by a reemergence of debates on the legitimacy of BLM in France.^11^ All presented analyses were pre-registered and available at: Pre-registration Study 2.

### Power Analysis and Sample Size

We ran two pre-registered power analyses for the mediation model and the path analysis using Monte Carlo simulations. For both, we set the parameter values for each path based on the coefficients of Study 1. Analyses were run with different *n* values (ranging between *n* = 300 and *n* = 400) using the ‘simsem’ R package. These simulations showed that a sample size of 400 is sufficient to reliably estimate the previously found regression coefficients for both analyses with a minimum power of 80%. We set the minimum sample size to at least 450 participants to compensate for potential exclusions.

In total, 569 participants took part in the study implemented in Qualtrics software and disseminated via social media. The study was conducted about one year after the previous one, in accordance with the research aim (i.e., June 15–25, 2021). We excluded participants who had completion times below three and a half minutes (*N* = 7),^12^ did not report French Nationality (*N* = 10), did not complete their primary/secondary education in France (*N* = 12), reported both parents’ mother tongues other than French (*N* = 10) or related to North- and West-African tongues (*N* = 25), had missing data on parents’ mother tongue (*N* = 10), or were minors (i.e., first-year students under 18; *N* = 7). We thus analyzed the data of the remaining 489 participants: 395 women, 88 men, and 5 non-specified (age range from 18 to 89 years old; *M_age_* = 33.95; *SD_age_* = 15.69).

### Materials and Procedure

The study was conducted following the same procedure and using the same R scripts as in Study 1. All materials, data and complementary material can be found at: supplementary material.

#### National Identification

The four items (Badea, 2012) were collapsed into a single National Identification index (*α* = .70).

#### Social Dominance Orientation

The short 8-item SDO scale (Duarte et al., 2004; Ho et al., 2015) was averaged into a single index (*α* = .85).

#### Laïcité Endorsement

The 13 items from Roebroeck and Guimond (2017) were submitted to an EFA analysis. Bartlett’s test of sphericity and the KMO statistic confirmed that the correlation matrix was not random (*p* < .001), and sampling was adequate (> .60). The overall scale has a good internal consistency (*α* = .73). Parallel analysis and the visual scree test suggested a two-factor solution with loadings consistent with the Historic and New Laïcité dimensions (*r* = -.06). However, we excluded one New Laïcité item that loaded on the wrong factor (see supplementary material for details). Thus, five items composed the New Laïcité index (*α* = .81), and seven items composed the Historic Laïcité index (*α* = .83).^13^

#### BLM Support

The three original items (Selvanathan et al., 2018) were adapted to measure generic BLM support: ‘In general, to what extent do you support the Black Lives Matter movement and the fight against racism in France?’; ‘During significant events (protests, lethal police violence etc.), to what extent would you be prepared to display your support for Black Lives Matter and the fight against racism on social networks’, and ‘How likely are you to support anti-racism protests in France in the future?’. The items were averaged into a single BLM support index (*α* = .87).

#### Sociodemographic information

Participants indicated their age, gender, mother tongue and their parents’ mother tongue, whether they had completed their education in France, their nationality and political orientation.

### Results

Means, standard deviations, and correlations among all measures in study 1 are presented in Table 2.

**Table 2:** Means, standard deviations, and inter-correlations between the main variables in Study 2.


VARIABLE	*M*	*SD*	1	2	3	4

1. National Identification	4.96	1.19				

2. SDO	2.47	1.13	.29***			

3. Historic Laïcité	6.17	0.94	–.10*	–.44***		

4. New Laïcité	4.85	1.48	.32***	.25***	–.08	

5. BLM	3.35	1.11	–.32***	–.58***	.45***	–.42***


*Note*: *N* = 489 * Indicates *p* < .05, ** *p* < .01, *** *p* < .001.

#### Mediation Analysis

**Figure 3:**
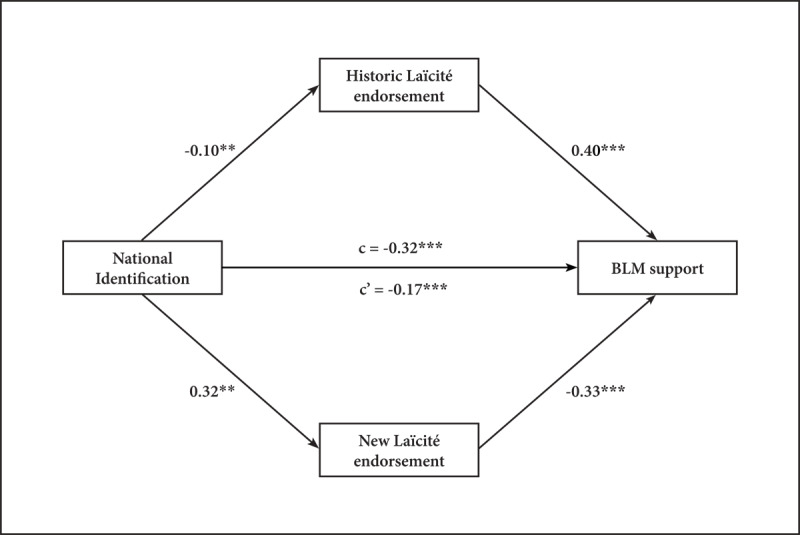
Replication of the mediation model of National Identification on BLM support via the endorsement of Laïcité norms. *Note*: Coefficients are standardized in our sample (*N* = 489). *c* represents the total effect and *c*’ the direct effect of NI on BLM support. * Indicates *p* < .05; ** *p* < .01; *** *p* < .001.

As expected, the analysis replicates the dual-mediation (see Figure 3). National Identification negatively predicts BLM support, *b* = –0.17, *t*(485) = –4.36, *p* <.001, through both the Historic Laïcité endorsement, *b* = –0.04, 95% CI [–0.08, –0.01] and New Laïcité endorsement, *b* = –0.11, 95% CI [–0.15, –0.07]. Moreover, all the component paths of both indirect effects are significant: National Identification predicts Historic Laïcité endorsement, *b* = –0.10, *t*(487) = –2.30, *p* = .02 and New Laïcité endorsement, *b* = 0.32, *t*(487) = 5.64, *p* < .001, and Historic Laïcité endorsement, *b* = 0.41, *t*(485) = 11.25, *p* < .001 and New Laïcité endorsement, *b* = –0.33, *t*(485) = –6.54, p < .001 predict BLM support.

#### Path Analysis

##### Hypothesized Model

**Figure 4:**
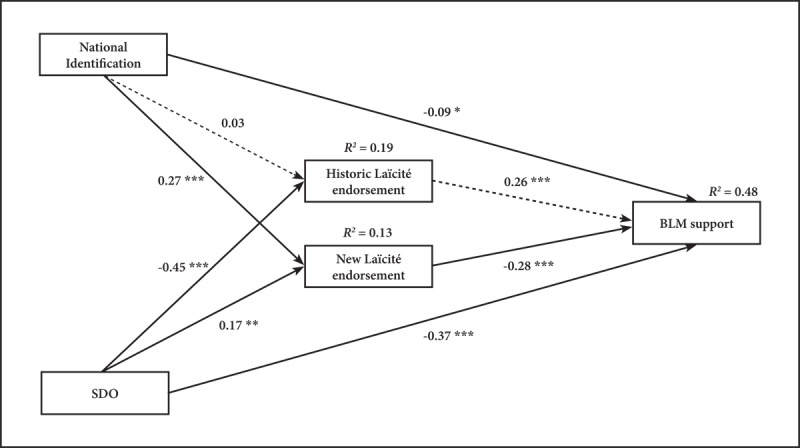
Replication of the path analysis including nationally-grounded and cross-national paths predicting BLM support. *Note*: Path coefficients are standardized, and R^2^ indicates explained variance. Full lines represent significant paths and broken lines represent nonsignificant paths. * *p* indicates < .05; ** *p* < .01; *** *p* < .001.

The hypothesized model provided excellent fit to the data: CFI = 1.00, TLI = 1.01 and SRMR = 0.01, RMSEA = 0.00, 90% CI [0.00, 0.11], with AIC = 4014.43. The model accounts for 48% of the variance of BLM support (R^2^ = 0.47). The analysis replicates and extends the path analysis from Study 1 (Figure 4). As in Study 1, BLM support is predicted by National Identification (*b* = –0.09, *p* = .01, 95% CI [–0.16, –0.02]), SDO (*b* = –0.37, *p* < .001, 95% CI [–0.44, –0.30]), New Laïcité endorsement (*b* = –0.28, *p* < .001, 95% CI [–0.34, –0.21]) and this time also by Historic Laïcité endorsement (*b* = 0.26, *p* =.001, 95% CI [0.19, 0.35]). Moreover, the New Laïcité is predicted both by National Identification (*b* = 0.27, *p* < .001, 95% CI [0.19, 0.35]) and SDO (*b* = 0.17, *p* < .001, 95% CI [0.09, 0.26]). However, mirroring Study 1, the Historic Laïcité is predicted by SDO (*b* = –0.45, *p* < .001, 95% CI [–0.52, –0.38]), but not by National Identification (*b* = –0.03, *p* = .48, 95% CI [–0.05, 0.11]). Concerning the indirect pathway, the one stemming from National Identification, via the New Laïcité endorsement, to BLM support is significant (*b* = –0.08, *p* < .001, 95% CI [–0.11, –0.05]), but not the one via Historic Laïcité endorsement (*b* = 0.01, *p* = .49, 95% CI [–0.01, 0.03]). The indirect pathway stemming from SDO, via the New Laïcité endorsement, to BLM support is also significant (*b* = –0.05, *p* < .001, 95% CI [–0.07, –0.02]), and in this study, the one via Historic Laïcité is also significant (*b* = –0.12, *p* < .001, 95% CI [–0.15, –0.08]). Additionally, there is no difference between the SDO-New Laïcité (*b* = 0.17) and the National Identification-New Laïcité coefficients (*b* = 0.27), W = 2.06, *p* = .15.

##### Alternative Models

We performed comparisons with the same two alternative models. Neither Alternative model 1: CFI = 0.95, TLI = 0.76 and SRMR = 0.06, RMSEA = 0.16, 90% CI [0.11, 0.22] with AIC = 7064.40, nor Alternative model 2: CFI = 0.92, TLI = 0.59 and SRMR = 0.10, RMSEA = 0.21, 90% CI [0.16, 0.26] with AIC = 7082.6, fit the data well. What is more, Vuong’s test indicates that the Hypothesized model is distinguishable from Alternative model 1(w2 = 1.13, *p* < .001) and Alternative model 2 (w2 = 1.15, *p* < .001) and fits the data better than both models (respectively, z = 64.67, *p* < .001; z = 64.66, *p* < .001).

### Discussion

In Study 2, we reasoned that National Identification via Laïcité endorsements are stable predictors of BLM support in France across time. Thus, we conducted a replication in France one year after the death of Georges Floyd. Notably, the findings replicate the dual-mediation and the pathways from National Identification and SDO, via the endorsement of New Laïcité, to BLM support. Moreover, in this replication, the path from Historic Laïcité endorsement to BLM support emerged as significant. One possible explanation is that, as compared to Study 1, the reliability of the Historic Laïcité index is somewhat higher (i.e., Study 1: *α* = .68 vs. Study 2: *α* = .81). Interestingly, the indirect path linking SDO to BLM support, via Historic Laïcité endorsement, is also significant. Yet, here again, the indirect path starting from National Identification is not reliable.

## General Discussion

The BLM protests stood as explosive international catalysts in the global fight against racism and police violence back in spring 2020. In this contribution, we examine whether nationally-grounded determinants also shape BLM support (Guimond et al., 2013; Weldon, 2006). To investigate this issue, we took France as a prime example. Indeed, this nation promotes a national identity which is blind to ethnocultural membership (Beaman & Petts, 2020). Further, two paradoxical cultural norms are used to regulate the displays of ethnocultural particularism: the Historic Laïcité, an egalitarian norm and the New Laïcité, an assimilationist one (Lankester & Alexopoulos, 2021; Roebroeck & Guimond, 2017). We reasoned that due to its shared content, French National Identification will negatively predict BLM support. Further, we proposed that Laïcité endorsement dually mediate this relationship (Adam-Troian et al., 2019; Badea, 2018; Pehrson et al., 2009). To strengthen our claim, we also included in our model an additional path starting with a cross-national predictor: SDO. We predicted that both pathways will contribute unique variance in BLM support.

Overall, the dual-mediation findings supported our rationale. In Study 1, at the peak of the BLM protest, the more French are national identifiers, the more they favor the New Laïcité and the less they endorse the Historic Laïcité which, in turn, leads to less BLM support. Study 2 conducted one year later and using a different sample replicated these results. Thus, National Identification and Laïcité norms seem to be relatively stable predictors of how the French take a stance on BLM. In fact, our contribution provides novel insights on how cultural norms function as mediators between National Identification and support for minority protests. Thus, beyond the French context, these results suggest that nationally-grounded factors—defining the ‘who’ and ‘how’ of national membership—may shape variations in BLM support in different nations. To grasp this national-sensitivity, future research should include comparative designs including nations that differ in their core definition of national identities and cultural norms. Moreover, using path analysis, our hypothesized model provides a better relative fit for the data than the alternative models. As predicted, we found, in both studies, that National Identification and SDO—via the endorsement of *New Laïcité*—contribute distinctly to BLM responses. In line with our hypotheses, these results provide evidence that the nationally-grounded path contributes to shape local responses to BLM in tandem with the cross-national one (Guimond et al., 2013).

However, in both studies, when adjusting for SDO in the path analysis, National Identification does not predict Historic Laïcité endorsement. This might be explained by the fact that we did not specify any covariation between SDO and National Identification in our path model (see, Footnote 7). Yet, there is empirical evidence that these two constructs can jointly mold diversity responses (Hindriks et al., 2014; Morrison & Ybarra, 2008). Consequently, French endorsement of Historic Laïcité could be explained by their joint operation. Another plausible explanation to investigate further is the operation of a third variable. For instance, perceived outgroup threat is a key mechanism connecting National Identification to specific cultural norm endorsement (Badea et al., 2018; Verkuyten, 2009). Furthermore, the path from Historic Laïcité to BLM support is unreliable in Study 1 but is significant in Study 2. We suspect that in Study 1, the error in this construct’s measurement resulted in an underestimation of its path coefficients (Cole & Preacher, 2014). First, in both studies, and replicating past results, SDO negatively predicts Historic Laïcité endorsement (Roebroeck & Guimond, 2017). Second, the findings of Study 2 match past studies showing that support for Historic Laïcité shapes positive diversity responses (for a review, see Lankester & Alexopoulos, 2021). Therefore, the pattern of findings in Study 2 is consistent with SDT theory: Historic Laïcité acts as a legitimizing myth insofar it mediates the SDO-diversity response relationship (Roebroeck & Guimond, 2017). In fact, as a further contribution, our studies are the first (to our knowledge) to provide a comprehensive analysis of both Laïcité as legitimizing myths. Yet, we found no evidence that National Identification is a more potent predictor of Laïcité endorsement than SDO. Ultimately, this supports the idea that cultural norms are functional to serve distinct motives (i.e., linked to identity and group-hierarchy concerns) rather than one specific psychological construct (Adam-Troian et al., 2019; Roebroeck & Guimond, 2017).

Yet, the correlational nature of our data invites us to caution, and our studies have limitations. First, to examine the role of nationally-grounded variables, we approached National Identification from a macro-societal analysis (see Footnote 1). Thus, we operationalized National Identification as the level of self-affiliation with this identity (Staerklé et al., 2010). However, future research may focus on a more fine-grained inter-individual analysis of the specific facets of (French) National Identification (e.g., civic-nationalism, ethnic-nationalism; Roccas et al., 2006; Smith, 2001) that predict BLM support. Moreover, a future contribution could also test our hypothesized model against the addition of other (cross-)national predictors. For instance, there is evidence that the negative impact of National Identification on outgroup responses can be partially accounted for by collective narcissism (i.e., the emotional investment in an unrealistic belief about the unparalleled greatness of an in-group; Golec de Zavala et al., 2009). Second, to assess BLM support we selected existing items, already used in the US context (Selvanathan et al., 2018). This choice was guided by meta-scientific considerations, fostering greater comparability and conceptual replications of research findings. Arguably, this measure remains global and generic. It also contains an item related to the support for anti-racist protests. Yet, future research should delve deeper into the BLM dimensions such as BLM’s claims (e.g., anti-racism), methods (e.g., collective action), or social impact (i.e., systemic challenge). As such, it could further address which specific facets of the movement are involved in its rejection by national identifiers. Finally, our studies would have gained in predictive strength by delineating the French participants based on their ethnic group belonging (Gale et al., 2021; Holt & Sweitzer, 2020). However, in France, the term ‘race’ was removed from the French constitution. Thus, such an investigation remains to this day quite challenging. Nevertheless, our integrative attempt emphasizes the significance of modeling the influence of the national context to comprehend the ebb and flow of diversity responses, such as a global surge against racial inequalities.

## Footnotes

In this article, to account for the composite relationship between National Identification and diversity responses we start from a macro-societal analysis, acknowledging that national identity content differs across countries (Hopkins, 2001; Pehrson et al., 2009; Weldon, 2006). In fact, the content of the shared national identity can be determined objectively using The Migrant Integration Policy Index (i.e., MIPEX; https://www.mipex.eu/access-nationality). To capture this aspect, we used generic items without any preliminary definition of National self-affiliation (see Badea, 2012). Yet, in the literature, another approach considers starting from an inter-individual analysis. Indeed, for a specific country, there are different modes of National Identification (e.g., nationalism and patriotism; Roccas et al., 2006; Smith, 2001). Yet, we do believe that these two approaches are not mutually exclusive but rather complementary. For instance, the modes of National Identification could moderate the link between National Identification and diversity responses (Pehrson et al., 2009).

This inclination of French national identity is particularly promoted since 2007. Indeed, successive governments have emphasized the need to curb the ‘identity crisis’ imputed to the (supposed) suboptimal integration of North-African and Sub-Saharan immigrant descendants (Ocak, 2016; Simon, 2012, 2013). For more detail on the French national identity content see: https://www.mipex.eu/ France.

It is important to distinguish diversity ideologies (i.e., belief systems regarding the ways society should approach diversity; Levin et al., 2012; Wolsko et al., 2000) from cultural norms of diversity (Guimond et al., 2013). Individuals can favor and endorse various diversity ideologies beyond their national boundaries. However, to count as a cultural norm, a diversity ideology must be the dominant socio-political standard within a given country (e.g., favored and supported by legal policies; Berry, 2006; Guimond et al., 2013). The latter are particularly predictive of outgroup responses (Anier et al., 2018; Guimond et al., 2013; Lankester & Alexopoulos, 2021).

We applied the Median Absolute Deviation method (MAD; Leys et al., 2019) on time completions (MED = 6.48, +/– 3 MAD). On the lower boundary, we excluded 5 observations, based on the minimum estimated completion time (total number of items = 30 items; minimum time to complete one = 5 sec). On the upper boundary, the analysis suggests 22 observations as potential outliers (i.e., acceptable range of values [–1.63, 14.60]). However, findings remain unchanged with or without it and there is no serious reason to consider that they do not belong to the distribution. Thus, they were included in the final sample (Leys et al., 2019).

Research showed that group membership (e.g., majority vs. minority) impacts national belonging (Verkuyten & Martinovic, 2006; see also, Staerklé et al., 2010) and the type of cultural norms favored and endorsed (Berry, 2006), including Laïcité norms (Kamiejski et al., 2012). Thus, as North- and West-African descendants represent the main ‘visible’ minorities, we excluded them from the main analysis (Kamiejski et al., 2012a; Simon, 2012, 2013).

Participants were invited to respond to the following item: ‘French political attitudes are usually categorized on a spectrum that ranges from extreme left to extreme right. Where would you position yourself on this scale?’ on a scale from 1 = *Extreme left* to 9 = *Extreme right*. As pre-registered, the primary purpose of this item was to explore the correlation between Laïcité endorsement and Political Orientation for future research purposes. This is why it was not included in the main analyses. Still, the interested reader can find an exploratory analysis including this variable on OSF.

For instance, some authors consider that ingroup identification predicts the level of SDO (Hindriks et al., 2014; see also Morrison & Ybarra, 2008), whereas others assume that SDO shapes ingroup identification processes (Osborne et al., 2017). In any case, studies show that National Identification influences diversity responses beyond SDO (Verkuyten, 2009), including in France (Adam-Troian et al., 2019). Thus, following path analysis practices, we did not specify any relation between these two constructs (i.e., lack of strong theoretical assumptions; McDonald & Ho, 2002).

We relied on TLI and CFI (> 0.90), SRMR and RMSEA (< 0.08) and AIC (the lowest AIC indicate the best fitting model; Gana & Broc, 2018; Stage et al., 2004). However, some scholars invite to caution when using the RMSEA with low degrees of freedom (i.e., 4 in our model) and small sample sizes because rejection rates are high (Kenny et al., 2014; Taasoobshirazi & Wang, 2016). Finally, the Chi-square indicator was not used as it becomes uninformative with *N* > 200.

We also tested an exploratory alternative model: a third pathway starting from Political Orientation was added to our hypothesized model. Yet, this model has an overall poor fit (see, supplementary material).

They should operate as relatively stable determinants, provided no major sociopolitical events would occur in the meantime that could mold differently the scope or the definition of National Identification and the Laïcité.

For instance, during the UEFA European Football Championship (i.e., June 11, 2021–July 11, 2021) several football teams took the knee in support of BLM. While the French team initially considered to do the ‘Knee protest’, they backed out at the last minute, leading to lively debates (Euronews, 2021).

The MAD method (Leys et al., 2019) was again used on time completions (MED = 407 sec, +/– 3 MAD). On the upper boundary, 43 observations were suggested as potential outliers (i.e., acceptable range of values [20.04; 793.95]). However, because the results remain unchanged, we kept them in the final sample (Leys et al., 2019). Yet, on the lower boundary, we excluded seven observations based on the same criterion as Study 1.

As recommended by Cole and Preacher (2014), to increase construct reliability of the Historic Laïcité index, we added four newly developed items. However, as pre-registered, insofar as the Historic Laïcité index’s alpha is satisfactory, and the objective was to carry out a replication of Study 1, these new items were not included in the main analyses. Yet, the findings remain unchanged, whether we include them or not in the Historic Laïcité’s index (see complementary analyzes at supplementary material).
